# Large Epidemiological Influenza A Outbreak in a Teaching Hospital from Guatemala City

**DOI:** 10.5402/2012/638042

**Published:** 2012-08-01

**Authors:** Carlos Mejía, Monica Silvestre, Iris Cazali, Judith García, Ruth Sánchez, Leticia García, Leticia Castillo, Ingrid Escobar, Sandra Terraza

**Affiliations:** Clínica de Enfermedades Infecciosas, Hospital Roosevelt, Guatemala City 01011, Guatemala

## Abstract

*Objective*. To describe the characteristics and interventions to control a large epidemiological Influenza A Outbreak. *Methods*. During the months of February to April 2006, a large outbreak of Influenza A was detected, which affected Health Care Workers and hospitalized patients in a large teaching Hospital in Guatemala City. Interventions to interrupt transmission were implemented and included barrier methods (N95 masks, respiratory isolation measures, etc.) and enhanced hand hygiene, vaccination of healthy Health Care Workers (HCW), restrictions for patient visits. *Results*. From February to April 2006, 59 hospitalized patients diagnosed with Influenza A. 19 AIDS patients (mortality: 71%) and 5/40 (12.5%) in other diseases: cancer (3), severe cardiac failure (1) and severe malnutrition (1). The attack rate at day 20 in doctors and medical students was 21% while in other HCW it was 10.5%. Within 3 weeks of the beginning of the plan, deaths were stopped and no more cases in HCW were detected after 3 additional weeks. *Conclusion*. A rapid, comprehensive plan for the control of nosocomial epidemic Influenza A outbreaks is essential to limit severe morbidity and mortality in hospitals who attend large immunocompromised populations, including AIDS patients. HCW regular vaccinations programs are mandatory.

## 1. Introduction

Influenza A and B cause highly contagious, acute respiratory illness, associated with high morbidity and mortality among persons with underlying diseases. Influenza can cause extensive nosocomial outbreaks with high mortality rates. The causes of death in the more severe forms of influenza are (a) severe pneumonia with respiratory distress, (b) neurological complications, and (c) secondary bacterial pneumonia. Outbreaks in children units, senior facilities, cancer centers, neonatal units, pulmonary rehabilitation centers, emergency departments, and others have been described. Large nosocomial outbreaks in large tertiary referral hospitals that provide care to large numbers of patients with advanced AIDS have not been frequently described. Roosevelt Hospital is a large teaching, university tertiary referral hospital in Guatemala City, with 850 beds capacity.

More than 3,500 AIDS patients were in followups, and 1561 treated with antiretroviral drugs at the time when the outbreak was detected in March 2006; all of whom were at potential risk to develop severe forms of Influenza. 35% of the patients have less than 50 CD4 cells/mm^3^ and 68% less than 200 CD4 cells/mm^3^, at the time the HIV infection diagnosis is made at Roosevelt Hospital.

The main admission diagnosis in the Internal Medicine Department in the men wards is AIDS (on average 25/66 beds are occupied by patients with AIDS). Throughout the country mortality rate at the time of the first hospital admission in these advanced AIDS patients has decreased from 85% to 89% in the pre-HAART era (1990–2000), to 18%–21% after the year 2000.

Rapid dissemination of Influenza virus is more likey in closed areas with poor ventilation (air exchanges less than 6 per hour), no policies for health care worker vaccination and suboptimal awareness about the clinical presentation of severe influenza in immunocompromised persons may contribute to the magnitude of the outbreaks.

The mortality rates in immunocompromised patient can be high, as we observed in this group of patients, with fulminant pneumonia and respiratory distress as the final cause of death.

## 2. Methods

During the months of February to April 2006, a large outbreak of Influenza A was detected at Roosevelt Hospital; health care workers and hospitalized patients were affected in this large teaching Hospital in Guatemala City. During the first 5 days of the initial suspicion of a possible respiratory outbreak, after 3 consecutive deaths in AIDS patients who were stabilized for different opportunistic infections, the first results of nasopharyngeal swabs were positive for Influenza A virus, with no other pathogens found. The patients had been hospitalized for diagnosis and treatment, developed acute respiratory failure, and died within 18–36 hours from the beginning of symptoms; testing for Legionella, rapid antigen test for Influenza A, Influenza B, and Parainfluenza virus in regional or national labs, and cultures for common bacterial causes for nosocomial pneumonia in our Microbiology Laboratory were negative.

An extensive triage and surveillance program in health care workers (HCWs) was initiated. We could establish that the medical students and the internal medicine residents in the ward had a clinical diagnosis of Influenza. We began to test HCW and patients in all Internal Medicine wards and 3 days after in all wards of the hospital. An algorithm for HCW management was rapidly implemented as follows.

Influenza diagnosis was established by clinical case definition and rapid test for Influenza A; viral cultures and histopathological examination of tissues from dead patients were also instituted. Patients were considered as having nosocomially acquired infection if they had been admitted at least 72 hours before the onset of the symptoms. Clinical triage was established in the Emergency room for the early detection of new Influenza cases from the community.

Interventions to interrupt the transmission were implemented and included

- barrier methods (N95 masks, respiratory isolation measures, etc.) and strengthening of hand hygiene,
- vaccination of healthy health care workers (HCWs) and furloughing of sick HCW for 3 to 5 days,
- antiviral use for treatment or prophylaxis of hospitalized immunosuppressed patients, initially with amantadine, and after several failures, and the results of susceptibility tests obtained from collaboration with the US CDC, with oseltamivir,
- daily active surveillance for early detection of Influenza cases in patients and HCW,
- daily active surveillance for early detection of Influenza A cases in new patients before admission from the emergency rooms,
- No family visits to the internal medicines wards were permitted for 3 weeks,
- No elective surgery was permitted during the last 10 days of the outbreak.

## 3. Results

Patient 1 presented during the first week in March with acute respiratory failure and died within 24 hours. His diagnosis prompted a reevaluation of three recent deaths; all of whom tested positive in histopathology, resulting in the recognition of the outbreak.

In the period from February to April 2006, 59 hospitalized patients were diagnosed with Influenza A; 19 of them were AIDS patients (mortality rate: 71%) while 5/40 (12.5%) had other underlying diseases including cancer (3), severe cardiac failure (1), and severe malnutrition (1). The final attack rate at one month was 21% in doctors and medical students and 10.5% in other HCWs (Figures 3 and 4).

**Figure 1:**
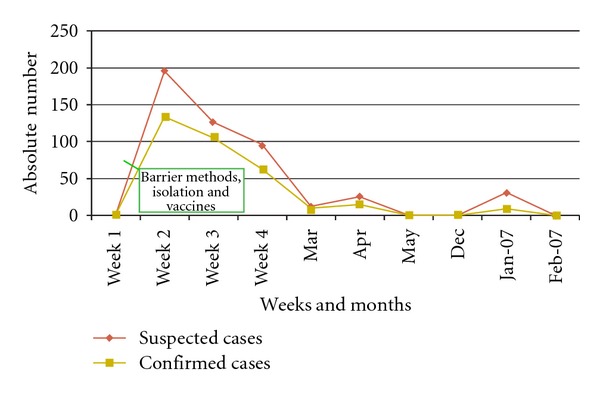
Epidemic curve of Influenza cases in health care workers (Mar. 2006–Feb. 2007), Roosevelt Hospital.

**Figure 2:**
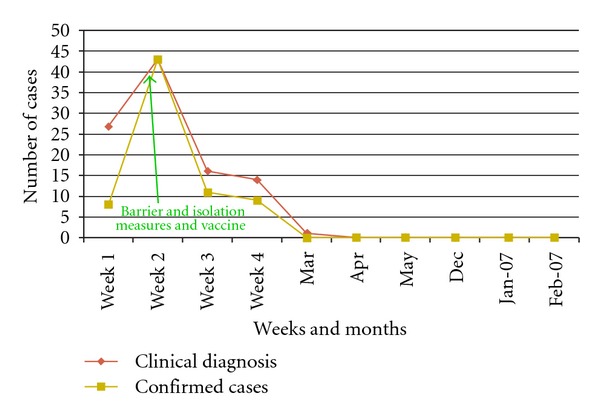
Epidemic curve, Medical doctors and students: Influenza cases (Mar. 2006–Feb. 2007), Roosevelt Hospital, Guatemala.

**Figure 3:**
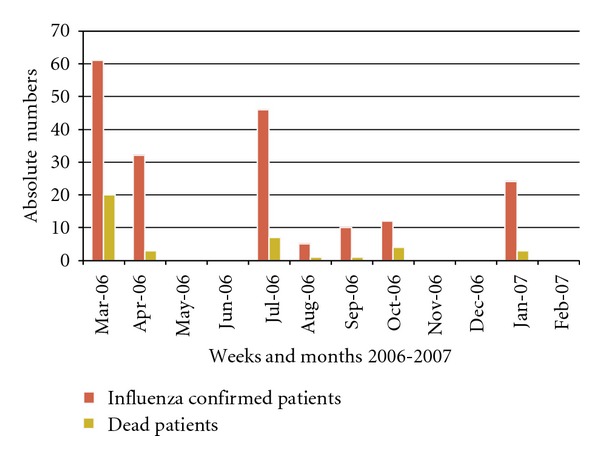
Total and confirmed Influenza cases on inpatients (Mar. 2006–Feb. 2007), Roosevelt Hospital, Guatemala.

**Figure 4:**
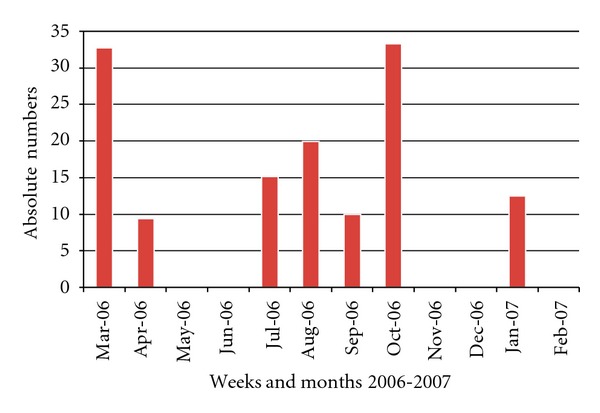
Lethality rate in Influenza patients (Mar. 2006–Feb. 2007), Roosevelt Hospital, Guatemala.

- A selective emergency vaccination plan was instituted in 5 days: 1,720 of 3,100 HCWs (56%) were reached (Figure 1).
- A daily active surveillance plan for early detection of Influenza cases in hospitalized patients and HCW was implemented.
- After 3 weeks of the implementation of the control plan, deaths were stopped, and after 3 more weeks no more cases were detected in HCW (Figure 2).

## 4. Discussion

Influenza A can cause a very severe disease, with a high mortality rate in advanced AIDS patients. The clinical presentation was characterized by the accelerated evolution to death from the beginning of symptoms (average 50 hours) and affected mainly the more advanced AIDS cases, with a mortality rate among this group of 74%, compared with the 12.5% mortality in the non-AIDS patients.

It is important to consider the possibility of Influenza in the differential diagnosis in advanced cases of AIDS that are admitted to the general internal medicine wards, who are under treatment for opportunistic infections and develop acute respiratory distress. The rapid evolution to death and the risk of transmission to other immunosuppressed patients can be stopped, if we implement general isolation measures and use antiviral drugs like oseltamivir as prophylaxis or therapy early in the course of the disease. The protection conferred by the available vaccines is uncertain, and the information in the AIDS population is limited.

All patients had concomitant severe opportunistic infections, but the histopathological findings showed in more than 90% of the patients in whom an autopsy was performed the typical pathology caused by influenza virus in the respiratory epithelium previously described. Amantadine failed to protect patients exposed to Influenza in the hospital, due to the resistance that was documented during the evolution of the outbreak. The Influenza A virus in advanced AIDS patient could be considered an acute and severe opportunistic infection, that requires rapid actions to limit the negative impact in morbidity and mortality in this population.

In addition to these points, several aspects of this outbreak are unique, and they deserved a discussion/comment. Few outbreaks of severe influenza have been described in tropical or subtropical regions; the fact that this one follows the seasonal pattern of the northern hemisphere season is worthy of a comment. This is one of the largest outbreaks in hospitalized patients with advanced AIDS and highlights the high risk of severe disease and mortality in these patients. Several aspects of the control plan instituted have been described as potential control measures for the control of outbreaks. Respirators, respiratory precautions, hand hygiene, and early diagnosis and furloughing of health care workers have been described. This plan shows that institution of all the measures may be necessary to control outbreaks. Vaccination of health care workers has been advocated but only mandatory programs have achieved 100% compliance. It would be good to know what the rate of vaccination in health care workers in the years after the outbreak has been. It is very urgent to investigate about the role of vaccination in the control of this outbreak, since it takes a few weeks to develop immunity after vaccination. The role of oseltamivir treatment and prophylaxis in the control of this outbreak also deserves some comment.

## 5. Conclusions

A rapid, comprehensive plan for the control of nosocomial epidemic Influenza A outbreaks is essential to limit severe morbidity and mortality in hospitals who attend large immunocompromised populations, including AIDS patients.

A regular vaccination plan for HCWs who serve debilitated patients is mandatory. A multidisciplinary approach is important to prevent possible future outbreaks, especially with increasing reports of human infection with highly pathogenic avian Influenza viruses or other novel viruses that could become pandemic in the near future.
